# From Bench to Field: A Guide to Formulating and Evaluating Anti-Tick Vaccines Delving beyond Efficacy to Effectiveness

**DOI:** 10.3390/vaccines9101185

**Published:** 2021-10-15

**Authors:** Charles Ndawula

**Affiliations:** 1 National Agricultural Research Organization, P.O. Box 295, Entebbe, Wakiso 256, Uganda; ndawulaj821@gmail.com; 2 National Livestock Resources Research Institute, Vaccinology Research Programme, P.O. Box 5704, Nakyesasa, Wakiso 256, Uganda

**Keywords:** anti-tick vaccine, anti-tick vaccine efficacy, anti-tick vaccine effectiveness, anti-tick vaccine protection

## Abstract

Ticks are ubiquitous blood-sucking ectoparasites capable of transmitting a wide range of pathogens such as bacteria, viruses, protozoa, and fungi to animals and humans. Although the use of chemicals (acaricides) is the predominant method of tick-control, there are increasing incidents of acaricide tick resistance. Furthermore, there are concerns over accumulation of acaricide residues in meat, milk and in the environment. Therefore, alternative methods of tick-control have been proposed, of which anti-tick cattle vaccination is regarded as sustainable and user-friendly. Over the years, tremendous progress has been made in identifying and evaluating novel candidate tick vaccines, yet none of them have reached the global market. Until now, Bm86-based vaccines (Gavac™ in Cuba and TickGARD^PLUS^™ Australia-ceased in 2010) are still the only globally commercialized anti-tick vaccines. In contrast to Bm86, often, the novel candidate anti-tick vaccines show a lower protection efficacy. Why is this so? In response, herein, the potential bottlenecks to formulating efficacious anti-tick vaccines are examined. Aside from Bm86, the effectiveness of other anti-tick vaccines is rarely assessed. So, how can the researchers assess anti-tick vaccine effectiveness before field application? The approaches that are currently used to determine anti-tick vaccine efficacy are re-examined in this review. In addition, a model is proposed to aid in assessing anti-tick vaccine effectiveness. Finally, based on the principles for the development of general veterinary vaccines, a pipeline is proposed to guide in the development of anti-tick vaccines.

## 1. Introduction

Ticks are ubiquitous blood-sucking ectoparasites capable of transmitting bacteria, virus, protozoa, and fungi to animals and humans [1,2]. Ticks are categorized into three families: Ixodidae (hard ticks), Argasidae (soft ticks), and Nuttalliellidae (monotypic ticks), of which the Ixodidae are the most significant with about 700 known species [3,4,5]. Of the three families, the argasid and ixodid ticks are of greater veterinary and medical importance [1,2]. However, more efforts have been devoted to the control of ixodid than to argasid ticks. This can mainly be attributed to the differences in feeding habits and, behaviors [6]. Additionally, in comparison to the two- or three-host ixodid ticks, the effectiveness of tick-control measures is likely to be higher against one-host ixodid ticks. Three-host ticks (e.g., *Rhipicephalus appendiculatus*, *Amblyomma variegatum*) are those that feed on a different vertebrate host at every stage of the life cycle, whereas one-host ixodid ticks (e.g., *Rhipicephalus microplus*, *Rhipicephalus decoloratus*, *Rhipicephalus annulatus*) feed on one host throughout their life cycle.

Until now, chemicals (acaricides) remain the frontline tool for livestock tick control [7]. However, the practical significance of acaricides is undermined by increasing acaricide tick resistance [8] and the concern over acaricide residue accumulation in meat, milk, and the environment [9]. For these reasons, researchers have proposed numerous alternative methods for livestock tick control [10]. Relative to the other alternative methods, the immunological tick control (anti-tick vaccination) is regarded as a user-friendly and sustainable treatment method [11]. Ideally, unlike with the two- or three-host ticks, three out of the four stages of the one-host tick life cycle (larvae, nymph, and adult), are pre-exposed to the mode of treatment, for instance, chemicals or anti-tick vaccine-induced antibodies. What this suggests is that, in comparison to the one-host ticks, the control of two- or three-host ticks would require a more expansive treatment approach in order to successfully interfere with the different stages of the tick life cycle. However, it remains unanswered whether, as with acaricides, a proportion of the tick population could acquire tolerance or resistance against anti-tick vaccines.

### What Is an Anti-Tick Vaccine?

**Figure 1:**
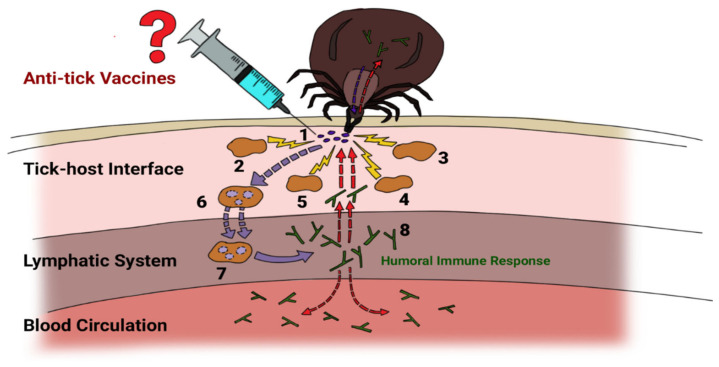
Tick-induced immune response: During feeding, ticks inoculate salivary proteins (1) which trigger recruitment of (2) antigen presenting cells (APC) which include macrophages and dendritic cells, (3) granulocytes which include neutrophils, basophils and eosinophils, (4) mast cells, (5) cytokines [16,17,18,19,20]. Salivary proteins are taken up by APCs (6), carried via the lymphatic system and presented to the lymph nodes to (7) induce a humoral immune response: antibody production (8). The antibodies move through the blood circulation and to the tick feeding site.

Anti-tick vaccines are tick proteins or peptides that induce host antibodies which confer protection against ticks. The anti-tick vaccine-constituting proteins (antigens) are broadly categorized into concealed and non-concealed antigens. Unlike concealed antigens, non-concealed antigens are derivatives of tick saliva proteins that are exposed to the vertebrate host during blood meal acquisition [12]. However, based on the above definition of anti-tick vaccines, one may unwittingly conclude that upon feeding on vaccinated cattle, the ticks will instantly be sterilized. On the contrary, unlike with the acaricides or insecticides, anti-tick vaccines do not instantly sterilize the ticks. In broad terms, anti-tick vaccines are defined as tick proteins which are administered to a vertebrate host to induce antibodies with potential to bind and interfere with or inhibit (A) the tick salivary proteins that are secreted at the host–tick feeding site to promote tick pathogen transmission and/or blood acquisition through modulation of the host hemostasis and immune responses, and (B) specific or related proteins that are expressed in the tick tissues to promote its biological parameters (tick feeding, egg laying, egg hatching, larvae, and/or nymph molting). Over time, vaccination against ticks leads to a reduction in the overall tick population and/or interferes with pathogen transmission, and therefore a reduction in the host–tick burden and tick-borne diseases. Specifically, the principle of anti-tick vaccination aims to mimic and exploit the immunological responses that transpire during routine tick feeding (Figure 1) toward affecting the tick biological parameters. Additionally, it should be noted that, potentially, some concealed (e.g., subolesin [13]) or non-concealed (e.g., 64TPR [14]) anti-tick vaccines interfere with pathogen transmission at the tick–pathogen or at the host–pathogen interface, respectively. The phenomenon can be explained through three possible mechanisms: the antibodies may (1) bind to tick proteins (e.g., midgut receptors proteins), which facilitate pathogen entry into the tick tissues for further development; (2) bind to tick salivary proteins that act a vehicle for pathogen entry at the host-tick feeding site; or (3) cross-bind to the pathogen proteins expressed at the host–pathogen and/or at the tick–pathogen interface.

Additionally, although some concealed and non-concealed anti-tick vaccines have shown potential to interfere or block tick–pathogen transmission, researchers have explored the possibility of developing more specific vaccines [15]. The more specific tick–pathogen transmission-blocking vaccines are developed based on the pathogen genes expressed at the pathogen–vertebrate and/or the tick–pathogen interface. What this means is that unlike anti-tick vaccine-induced antibodies which bind to the tick proteins and/or cross-bind to the pathogen, transmission-blocking vaccines induce antibodies specifically that bind to the pathogen protein(s) to interfere with its development in ticks and/or in the vertebrate host. Beyond that, it is thinkable that the tick–pathogen transmission-blocking specific vaccines may cross-bind to the structurally related tick proteins. Whether that can impact on the tick biological parameters, remains an open question.

Through the described mechanism (Figure 1), tick salivary proteins can promote acquired tick animal resistance [21]. Note that the vertebrate host resistance is influenced by other factors such as the animal species, animal breed and tick species [22,23]. Evidently, building on the host acquired resistance phenomenon, Trager [24] initiated the concept of anti-tick vaccines in 1939. However, although salivary proteins are regularly inoculated during tick feeding, the proteins are insufficient to instantly induce a strong immune response [25]. Therefore, the inoculation of an anti-tick vaccine aims to instantly induce a strong humoral immune response. However, since the inception of the concept of anti-tick vaccines [24], only two vaccine derivatives of the Bm86 antigen (Gavac™ in Cuba and TickGARD^PLUSTM^ in Australia) have been commercialized, of which TickGARD^PLUSTM^ is no longer available on the market. Even though Gavac™ has shown success in controlling *R. microplus* in Cuba [26], the vaccine is shown not to equally induce protection against the *R. microplus* strains of South America [27]. This is attributed to the genetic diversity of *R. microplus* [28]. Nonetheless, the vaccine was shown to induce cross-protection against other one-host ticks, *R. annulatus* [29] and *R. decoloratus*, but not against three-host ticks, particularly, *R. appendiculatus* and *A. variegatum* [30]. It is unclear whether the low cross-protection was attributed to the difference in vaccine pre-exposure time against the one-host ticks compared to the pre-exposure time against three-host ticks. Unfortunately, while examining the effect against three-host ticks, the vaccine was assessed against only one developmental stage of the tick life cycle (adults). Although progress has been made in identifying tick antigens, the question remains: why is it that the new anti-tick vaccines have not reached the market? [31].

The key observations when assessing new antigens are that the factors which influence vaccine efficacy are less scrutinized and that the vaccines fall short of the goal, that is, to reduce the overall tick population. Therefore, the premise of this article is two-fold: (1) to highlight the bottlenecks to formulating efficacious anti-tick vaccines; and (2) to propose a model for conducting anti-tick vaccine effectiveness studies and a pipeline for assessing the vaccines from the bench to the field.

## 2. The Basic Immunological Principle of Anti-Tick Vaccination

World-wide vaccines are classified into two groups: T-cell-independent and T-cell-dependent vaccines [32,33]. Specifically, in contrast to T-cell-dependent vaccines, the T-cell-independent antigens do not require helper T cells to induce adaptive immune responses. Consequently, T-cell-independent vaccines induce a shorter lasting adaptive immune memory compared to the T-cell-dependent vaccines [34]. The mechanism of immune memory induction is illustrated clearly with human vaccines [35,36], but not with anti-tick vaccines. Nonetheless, it is likely that the mechanism is similar with anti-tick vaccines. For a deeper insight on the basics of immune responses, refer to Abbas et al. [37] In regard to memory induction, highly immunogenic tick antigens such as Bm86 [38] and GSTs [39] are reported to have T- and B-cell epitopes. What this suggests is that after obtaining omics data, researchers should examine the immunogenic potential of the target protein(s) based on the presence of B- and T-cell epitopes [40]. Fortunately, this can easily be achieved using immunoinformatics tools. For instance, based on tick GST protein coding sequences, Ndawula et al. [39] proposed a pipeline for predicting B- and T-cell epitopes.

### Hypothetical Mechanism of Anti-Tick Immune Memory Induction

**Figure 2:**
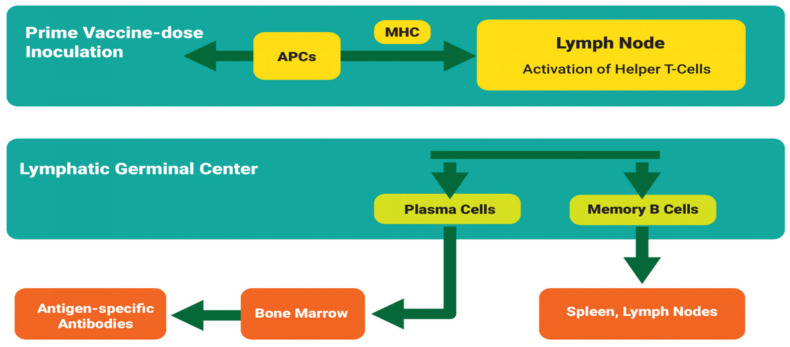
A schematic illustration of hypothetical mechanism of anti-tick immune-memory induction. Abbreviations: MHC (major histocompatibility complex). APCs (antigen presenting cells).

In brief, after inoculating the initial vaccine dose (priming dose), the antigen presenting cells (APCs), such as dendritic and macrophages [41], absorb, process, and present the antigen in the lymph node via the major histocompatibility complex (MHC) [42]. The activated dendritic cells interact with naive T cells which differentiate into effector T-helper cells. These cells migrate to the lymphatic germinal center where they interact with naive B cells which leads to the formation plasma and memory B cells. The plasma cells migrate to the bone marrow [43] leading to the production of antigen-specific antibodies, whereas the memory B cells sequester in the spleen and lymph nodes [44,45] whenceforth they await subsequent cognate antigen inoculation. The probable mechanism is illustrated in Figure 2. Refer to Abbas et al. [37] for further understanding of the fundamentals of basic immune responses.

## 3. Determination of Anti-Tick Vaccine Efficacy

Note that although the term anti-tick ‘vaccine efficacy’ is used interchangeably with ‘anti-tick vaccine effectiveness´ [46], the two terms are distinct [47] (vaccine effectiveness is discussed in Section 6). Vaccine efficacy is the measure of the vaccine performance under ideal controlled experimental house/laboratory conditions. In line with conventional vaccines [48], anti-tick vaccine overall efficacy is the proportional measure of reduction in the reproduction success of ticks fed on vaccinated experimental vertebrate hosts compared to those fed on unvaccinated hosts under ideal controlled conditions.

Mathematically, anti-tick vaccine overall efficacy (E) is the relative effect on ticks fed on vaccinated and to ticks fed on unvaccinated animals. Note that in principle, tick reproduction success incorporates the male tick fertilization and the entire tick life cycle. However, often the vaccine-induced overall effect is assessed based on the reduction in blood acquisition, weight and viability of eggs, and the number of female engorged ticks only [49].

$$E\%\;=\;100\;\times \;[\frac{Tcontrol-Tvaccinated}{Tcontrol}]\;=\;100\;\times \;[1-\frac{Tvaccinated}{Tcontrol}]$$

where *T_Vaccinated_* is the effect on ticks fed on vaccinated animals. *T_Control_* is the effect on ticks fed on control animals.

Currently, researchers use different methods to evaluate the efficacy of anti-tick vaccines [50,51,52]. For example, the formulae that are often used to assess the vaccine-induced effect on one-host and three-host ticks [50,51,52] only take into account the female tick engorgement, oviposition, and egg hatchability [49]. Although not often used, another formula has been proposed to assess the vaccine effect against three-host ticks [51]. In contrast, this method examines the effect induced on the weight and number of the ticks while at different stages of the life cycle [53]. What these differences indicate is that researchers are yet to assent on a uniform method of calculating anti-tick vaccine efficacy [54].

## 4. Bottlenecks to Determining Anti-Tick Vaccine Efficacy

Although the goal of the researchers is to attain anti-tick vaccines with a high vaccine protection efficacy, note that efficacy mainly depends on the induced humoral immune responses or antibodies. Therefore, hereafter we examine (1) the factors that could influence the antibody production, and (2) the limitations to the methods currently used to assess vaccine efficacy.

### 4.1. Determinants of Anti-Tick Vaccine Immune Response Induction

#### 4.1.1. Adjuvant Anti-Tick Vaccine Formulation

Currently, anti-tick vaccines are most often expressed in *Escherichia coli* than in *Pichia pastoris* [55]. The drawback of *E. coli* expression systems is that the resulting proteins are not likely to undergo proper folding and post-translational modification [56]; hence, affecting their immunogenicity and bioactivity. However, this does not suggest that all proteins expressed in *E. coli* systems are of improper structure, that they lack bioactivity, or that they are not immunogenic. Rather, this suggests that *E. coli* expression systems are not always ideal for the expression of all tick proteins. Nonetheless, it is vital that adjuvants are added to all anti-tick vaccine formulations to augment their immunogenicity. Adjuvants can enhance immunogenicity mainly through two mechanisms: (A) antigen depot formation to ensure slow release and induction of cell-mediated immune responses which are a key precursor in stimulating a strong humoral immune response [57], and (B) inert delivery of antigen to the target immune cells [57]. Some adjuvants could confer a dual function [58]. Further, adjuvants may improve stability and prevent degradation of the antigen [59]. Although advances have been made in adjuvant development [60], a few adjuvants, namely Complete Freund’s adjuvant (CFA), Incomplete Freund’s adjuvant (FIA), and montanides have been exploited in the formulation of anti-tick vaccines [55]. Note that all of these are emulsion-based adjuvants which act through depot formation at the injection site and slow antigen release to stimulate the immune system [61]. Yet, other forms of adjuvants, such as mineral compounds (e.g., alum, calcium salts), liposomes, bacterial products (e.g., bacterial derived toxins and non-toxins), ISCOMs, tensoactive compounds (e.g., Quil-A), and nanoparticles, have been reported [62]. Nevertheless, like with other vaccines, the selection of adjuvants for the formulation of anti-tick vaccines should be based on the adjuvant potency and the minimal induction of side effects [63]. However, reports on comparison studies regarding the effect of adjuvants on the efficacy of anti-tick vaccines are scarce [63]. Therefore, this could be a key factor, but certainly not the only reason for the paucity of commercial anti-tick vaccines.

#### 4.1.2. Vaccine Dose Administration

(A) Prime vaccine dose

**Figure 3:**
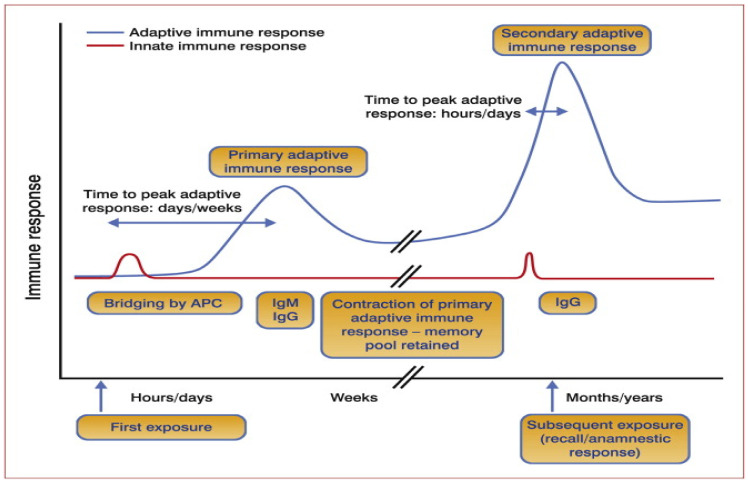
Illustration of the humoral immune response kinetics curve adapted from Leo et al. [104].

There is a consensus that the antigen dose impacts proportionally on humoral immune responses and the vaccine protection outcome [64,65]. However, a higher antigen dose may not necessarily be better [65,66,67]. For example, unlike with a low antigen dose, when primed with a high antigen dose, the antibody response is higher and quicker, but the antibody titer levels off earlier [68]. Additionally, in contrast to low-dose-induced antibodies, the antibodies due to a high antigen dose are of higher affinity/avidity, yet such antibodies impair the antibody formation mechanism after immunization [68,69,70,71]. In other words, low-affinity antibodies enhance the initial immune response and B-cell activation. By definition, affinity is the binding strength between the antibody epitope and the antigen binding sites (parotype), whereas avidity is the measure of total antibody complexes. Ultimately, inoculating a high antigen concentration also induces vaccine immunotolerance, which in return reduces vaccine efficacy [69,72,73]. The optimum inoculation dose could vary depending on (A) the type of antigen and its ability to trigger different components of immune system [74], (B) the adjuvant used in the vaccine formulation, whereby each adjuvant has unique characteristics and action mechanisms [75], (C) route of administration [60,76], and (D) host related factors. Therefore, with regard to anti-tick vaccine administration, the question is: does the vaccine dose concentration increase with body size? In principle, an equal vaccine dose should be administered irrespective of the size, body weight or breed [77,78,79]. So, how is the vaccine dose determined? Contrary to the principle of drug dose administration [80] (which depends on the circulating drug concentration in blood or tissues), vaccine dose determination depends on the number of circulating lymphoid molecules. Inoculating a vaccine dose based on animal body weight could lead to undesirable immunological effects, and hence a reduction in vaccine efficacy. Therefore, to accurately determine the optimum anti-tick vaccine dose it is important to exploit the humoral immune response kinetics curve (Figure 3) that is based on data derived from pilot-dose response immunological studies performed with a wide range of antigen concentrations. Alternatively, the prime vaccine dose may be determined based on existing information about similar antigens. For instance, data generated from previous studies on protective vaccines, such as for Bm86 [81], could be of use in determining dose concentration of membrane antigen-based anti-tick vaccines. In addition to assessing the antibody titer after inoculating the prime vaccine dose, researchers ought to investigate the antibody avidity [82], given that it depicts antibody affinity.

(B) Booster vaccine dose

The aim of boost vaccination is to elicit a specific, robust, and long-lasting immune protection. Note that although booster vaccination also follows the earlier-described action mechanism (Figure 2), the response is faster [83]. This is because the booster vaccine exploits the prime vaccine-induced memory B cells which change into plasma B cells to produce antigen-specific antibodies. However, evidence shows that the secondary immune response is inversely proportional to primary immune response [84,85]. For instance, Hanna and Peters [85] illustrated that priming an animal with a low vaccine dose leads to a stronger secondary immune response than priming with a higher vaccine dose. However, until now the optimum anti-tick vaccine prime dose is not known. Rather, often tick researchers administer the same vaccine dose during prime-booster vaccination. Based on the discussion regarding the effect of the prime vaccine dose on avidity and immune tolerance, two questions arise: (1) could it be that the administration of a high booster vaccine dose affects the avidity of the secondary antibodies, hence the anti-tick vaccine efficacy?; and (2) does administering half the prime vaccine dose as a booster vaccine dose confer a stronger humoral response? In other words, it is important that the above factors are taken into account when formulating efficacious anti-tick vaccines.

#### 4.1.3. Prime Boost Vaccination Strategy

The concept of prime boost dose anti-tick vaccination was conceived based on the observation that successive tick–animal feeding induces acquired immunity [24], hindering the subsequent tick batch from feeding to engorgement. What this observation suggests is that tick–animal infestation triggers immune memory (Figure 1). After inoculating hosts with tick larvae crude protein extracts, Trager [86] further observed antibody production. However, considering that subunit vaccines, (anti-tick vaccines inclusive) are less immunogenic [87,88], prime boost vaccination must be applied to ensure induction of a sufficient immune memory. There are two methods of prime boost vaccination:

(A) Homologous prime boost dose vaccination

This involves repeated host vaccination (priming and boosting) with the same type of vaccine or antigen [89]. The strategy is widely used for investigating vaccine efficacy. However, with regard to anti-tick vaccines, the strategy has shown mixed success. It is probable that the homologous prime boost vaccination strategy leads to a weak cellular immune response [90]. For instance, homologous prime boost vaccination tends to diminish release of antigen presentation cells and associated signals [89,90]. Therefore, it is likely that this strategy influences the efficacy of new anti-tick vaccines.

(B) Heterologous prime boost dose vaccination

This involves host repeated vaccination (priming and boosting) with different types of vaccines or antigens. For instance, DNA prime/protein booster vaccination [91,92]. This strategy has been adopted to circumvent the limitation of the homologous prime booster vaccination strategy. The benefit of heterologous prime boost vaccination is that it enhances cell-mediated responses, leading to a high humoral immune response [89,91,92,93,94,95,96] which influences vaccine efficacy [96]. However, with regard to anti-tick vaccination, the heterologous prime boost strategy is underexploited [97]. Yet, this vaccination strategy could enhance the efficacy of anti-tick vaccines.

#### 4.1.4. Vaccination Interval and Frequency

The underlying tenet of anti-tick vaccines is to elicit a long-lasting, robust adaptive immune response to limit the animal–tick burden [98]. Immune protection depends on the ability of the memory B cells to generate effective antibodies after inoculation with the cognate antigen [99]. In humans, when there is continuous immune system exposure to the cognate antigen that was used for priming, the protection can last for decades [96]. It is possible that a similar phenomenon could occur with anti-tick vaccines, particularly with non-concealed antigens [12] or concealed/non-concealed (those expressed in saliva and other tissues) [99]. However, the response could be slower considering that a minute quantity of salivary proteins are inoculated during tick feeding [18]. Furthermore, the serum immunoglobulins have a short half-life of 1–3 weeks [100,101]. Therefore, to sustain the immune response, it is necessary to inoculate booster vaccine dose(s) by taking into account two fundamental considerations:

(A) Vaccination booster frequency

Evidence shows that booster vaccination at a short interval after the priming does not lead to induction of a stronger secondary immune response, rather it interferes with the primary response, which leads to immune tolerance [102]. For that reason, while determining the interval for administering the booster dose, researchers should consider the humoral immune-response kinetics curve (Figure 3). For instance, in humans, the humoral immune-response self-termination feedback mechanism occurs at least 2–3 months after the primary vaccination, which is optimal to induce a robust secondary adaptive immune response [103]. However, delays in booster vaccination beyond 3 months does not guarantee improved secondary adaptive immune response [103]. The benefit of allowing the primary immune response to wane is that it allows the completion of affinity maturation and the development of highly specific memory B cells which would subsequently lead to a stronger immune response. For a deeper insight on affinity maturation, refer to Abbas et al. [37]. By contrast, often the booster anti-tick vaccine doses are administered after two weeks [55]. This suggests that most of the anti-tick vaccines are administered before the prime immune response has waned which could affect the immune memory induction, and hence the vaccine protection efficacy.

(B) Number of administered booster vaccine doses

With regard to booster vaccination, it is common practice for tick researchers to administer at least two doses [55]. It is presumed that the more anti-tick vaccination booster doses, the stronger the immune protection. However, practically, this does not occur. Therefore, it is likely that the commonly used booster vaccination approaches [55] lead to interference between the booster-vaccine responses, and hence reduced anti-tick vaccine efficacy. Beyond that, administering a high number of booster doses has vaccine cost implications. Unlike with the anti-tick vaccines, the booster dose of some human vaccines is administered after a longer interval. For instance, the tetanus and diphtheria vaccine booster doses are administered after ten years [105], whereas the interval for hepatitis B is at least 20 years [106]. However, with regard to anti-tick vaccines, reports about booster vaccination doses are scarce. For instance, based on Bm86 (Gavac^plus^) vaccination against *R. microplus*, Vagas et al. [107] demonstrated that one booster dose per year was sufficient to maintain humoral protection and that the booster dose should not be administered until the antibody titer drops below 1:640. Therefore, it is also possible that anti-tick vaccines, particularly those derived from proteins expressed in the tick saliva and other tissues, may induce a longer protection. The hypothesis is that after administering the prime dose of a concealed/non-concealed vaccine, regularly during blood ingestion, ticks will inoculate the cognate salivary proteins (antigens) in minute quantities, which will continue to sensitize the immune system, hence maintaining humoral immune responses.

Finally, it is prudent that while determining the number of booster doses, the researchers review the humoral immune response kinetics (Figure 3). Note that the booster dose could also be influenced by the booster vaccination strategy, route of administration, animal age, and the booster dose.

#### 4.1.5. Route of Vaccination

Worldwide, vaccines are administered through two routes: mucosally and parenterally. The mucosal route entails administering the vaccine via the mucosal membrane (e.g., nasal, rectal, ocular, oral, vaginal), whereas the parenteral route entails injecting the vaccine through the skin. The skin layers are highly populated with immune cells, such as the granulocytes, antigen-presenting cells, and cytokines, which are key components for the induction of antigen-induced innate responses; a sine qua non for induction of adaptive immune responses [108,109,110,111]. Therefore, to access the skin’s layers, parenteral vaccination can be executed via three routes: subcutaneous (SC), intradermal (ID), and intramuscular (IM) [112]. Of the three routes, the parenteral SC route is commonly used to administer anti-tick vaccines.

By contrast, other vaccines (for example, human vaccines) are commonly administered via the parenteral IM route. Generally, vaccination via the IM route induces higher and quicker adaptive immune responses than vaccination via the SC or ID route [113,114,115]. This phenomenon can be explained as follows: unlike via the ID or SC route, when vaccines are inoculated via the IM route, they drain directly into the lymphatic system [114]. In addition, IM vaccine inoculation induces fewer adverse effects than the SC route. For example, in comparison to the IM inoculation, often reactogenicity occurs at the site of injection after ID and SC inoculation [116,117]. Moreover, vaccination via the ID or SC route requires a higher dose compared to vaccination via the IM route [117,118,119]. To enhance the anti-tick vaccine immune protection, researchers may consider using the IM route for prime dose inoculation and other routes for booster dose inoculation. For instance, McCluskie et al. [120] illustrate that parenteral prime mucosal boost vaccination elicits a diverse immune response.

In addition to the parenteral route, recently, Contreras et al. [121] demonstrated the possibility of administering anti-tick vaccines via the mucosal route. Notably, rather than combining the subolesin with the commonly used emulsion adjuvants [55], the vaccine was combined with an immunostimulant: heat-inactivated *Mycobacterium bovis*. It was demonstrated that when anti-tick vaccines are administered via the oral route, it is possible to still attain a high protection efficacy while yet minimizing the stress inflicted to cattle during vaccination [121]. Simply, this is because via the mucosal route, vaccines are administered non-invasively, which does not require the use a of needle. The rationale is that, unlike the skin, which is stratified and keratinized, the mucosal membrane is comprised of a thin single-layered epithelia which permits rapid permeability of the vaccine to the underlying immune cells [122]. Although the mucosal route is attractive, administering anti-tick vaccines (immunogenic tick proteins) orally poses a risk of antigen/protein degradation due to digestive enzymes and bacterial proteases [123].

Nevertheless, to enhance the anti-tick vaccine animal immune protection, researchers may consider using the IM route for prime dose inoculation and other routes for booster dose inoculation. For instance, McCluskie et al. [120] demonstrates that parenteral prime and mucosal boost vaccination elicits a diverse immune response. Finally, it is worthwhile to further exploit the benefits conferred by adjuvants when combined with vaccines [118], and to remember that the route could be influenced by adjuvants [124,125].

#### 4.1.6. Vaccination Animal Model

Until now, naive small animal models (e.g., rabbits, guinea pigs, and mice) or large (sheep, and cattle) are used to assess the efficacy of anti-tick vaccines [55,91]. It should be noted that animal models exhibit varying immunocompetence, which significantly influences the vaccine-induced humoral response. The difference in immunocompetence is higher in mice [126] than in rabbits [127]. Similarly, different cattle breeds, exhibit variable immunocompetence and resistance against diseases or vectors [128,129,130]. The difference in resistance is attributed to the variation in T-cell responses among cattle breeds [130,131]. On the contrary, evidence shows that after anti-tick vaccination, no significant variation in humoral immune response was exhibited among African and European cross-bred cattle [53,132]. However, these studies did not investigate T-cell responses.

Aside from the animal breed, age influences the vaccine immune response. For example, in humans [133], the immune system wanes with age. By contrast, reports on whether the same phenomenon occurs in animals are scarce. Nonetheless, from the field point of view, the older the animal, the more it is exposed to ticks and tick-borne diseases, hence acquiring partial immunity. However, this does not imply that younger animals should be administered with higher vaccine doses than the older animals. The implications are elucidated above. Therefore, in an effort to ensure uniform protection among all animals, researchers ought to consider the following:

(A) The immunocompetence background of the animal model; (B) prime booster vaccine dose administration intervals—in comparison to younger animals, older animals may require a longer interval after the priming dose and between the booster doses; (C) route of administration; and (D) number of booster vaccine doses. Unlike older animals, younger animals may require regular booster vaccine doses. In summary, irrespective of animal breed or age, anti-tick vaccines should confer insignificant variations in the induced immune protection efficacy.

## 5. Approach of Assessing Vaccine Efficacy

In general, the current approach of assessing the efficacy of tick vaccines is based on one-host tick species. This entails determining the relative weight reduction of engorged female ticks, eggs, and viable larvae from vaccinated and non-vaccinated hosts [50,51]. However, using this approach, it is not possible to deduce whether the vaccine could reduce the tick population. For example, in the current protocols [50,51], after feeding on experimental animals, the weight of engorged female ticks is recorded to assess the vaccine effect on tick feeding success. In principle, the weight of the tick is directly proportional to the number of eggs [134,135]. Therefore, it is anticipated that ticks fed on vaccinated animals will ingest less blood compared to those fed on unvaccinated animals. It is expected that the ticks which fed on vaccinated animals will lay fewer eggs compared to the eggs from ticks which fed on the unvaccinated animals. Further it is anticipated that the eggs from the ticks of the vaccinated group may fail to hatch, or that the larvae hatched from the eggs of the ticks which fed on vaccinated animals will weigh less compared to the larvae hatched from eggs of the ticks which fed on unvaccinated animals. However, to establish the weight, the larvae are sterilized by freezing. The drawback to this approach is that the viability of the larvae hatched from the eggs from ticks which fed on vaccinated or unvaccinated animals cannot be examined (i.e., nymph development). With this approach, the following questions arise: will the larvae from eggs of lower weight imbibe less blood? If the answer is no, does that imply that larvae cannot molt into nymphs? Additionally, if the larvae do molt, what is the fate of the nymphs? In other words, using the current approach [50,51], it is not possible to extensively assess the anti-tick vaccine efficacy and effectiveness. Therefore, to develop effective universal anti-tick vaccines, there is need for a model for assessing population reduction and mortality of single- and multiple-host ticks. Herein, an alternative approach to determining anti-tick vaccine efficacy while considering the above questions is proposed.

### 5.1. Assessing the Anti-Tick Vaccine Efficacy against Three-Host Ticks

This model is based on *R. appendiculatus* which feeds on three different hosts throughout its life cycle. Note that although rabbits are mentioned in subsequent discussions, they are not the best model for vaccine efficacy studies for one-host ticks [136].

Step 1: Assess the humoral immune response kinetics

Collect serum after prime dose vaccine inoculation, before and throughout the tick infestation experiments. Preserve the serum at −20 °C. It will later be used to assess the vaccine induced humoral response kinetics. The assumption is that earlier independent vaccine immunogenicity studies were performed in rabbits or mice and that the different vaccine parameters were established. These are the vaccine dose, vaccination strategy, booster vaccination interval, and route of vaccine administration.

Step 2: Assess adult tick engorgement

Divide naive rabbits into two groups (one unvaccinated control and one vaccine treatment) each made up of at least three rabbits. Infest each rabbit with 60 adult ticks (30 males and 30 females) obtained from the same tick colony batch. Monitor treatment groups daily for 5–7 days [137]. Determine the number of ticks successfully attached after 24 h. Collect male and female ticks as they detach from the host after engorgement. Note the term ‘engorgement´ refers to when a tick has fed to repletion and detached from the host. Ticks can feed to partial or full engorgement. Determine the number and weight of female ticks that successfully fed to engorgement. Calculate the difference in weight between the female engorged ticks collected from the control and vaccine-treated rabbits. However, engorgement does not apply to male ticks. There are two reasons: (1) the male ticks ingest less blood than the female ticks. Rather, they feed to ensure copulation and promote female tick feeding [138]; and (2) The male ticks detach and reattach probably to copulate with different female ticks. It is possible, based on these data, to deduce whether the vaccine affects tick feeding, although this does not imply that the harvested ticks cannot lay eggs or that the eggs cannot hatch and develop into nymphs and adults.

Step 3: Vaccine effect on adult tick egg laying (oviposition)

Incubate the female ticks independently under the appropriate conditions [137]. Collect data on the number ticks laying eggs, and number/weight of eggs per tick.

Determine the number of eggs by weight

Weigh a portion of the eggs of at least three ticks of equal weight that were fed on the same rabbit. Count the number of eggs in each portion. The eggs can be counted under a magnifying glass. Return the counted eggs to the egg cluster and weigh the entire eggs per tick. Based on the proportionality between the total egg weight and the number of eggs, determine the total number of eggs in the entire egg cluster per tick. Calculate the average number of eggs from the three ticks. Weigh separately the eggs from the remaining ticks and, based on the tick weight and average egg count, calculate the number of eggs from each of the remaining ticks. Finally, calculate the average and standard deviation of the number of eggs per group. Take care not to damage the eggs while handling and counting.

Mathematical example:

Presume that the engorged tick 1, 2, and 3 each weigh 0.5 g.

A portion of eggs from tick 1, 2, and 3 weighs 0.1 g, 0.14 g, and 0.15 g and the corresponding egg number is 450, 480, and 500, respectively. The total egg weight cluster per tick is 0.31 g, 0.33 g, and 0.35 g.

Therefore, the total number of eggs per tick can be calculated as below:


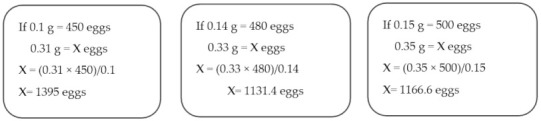


Batch average tick number: (1395 + 1131.4 + 1166.6)/3 = 1231 eggs.

Batch average egg weight: (0.31 + 0.33 + 0.35)/3 = 0.33 g.

Step 4: Vaccine effect on egg hatchability

(A) Independently pool and mix (e.g., using a paper) the eggs of ticks from each rabbit. Then, weigh a portion of eggs equivalent to the average cluster egg weight per tick (calculated in Step 2) and dispense the egg clusters into separate microtubes. Place the microtubes into separate bottles and incubate while slanted under appropriate conditions [137].
(B) After the eggs have hatched, expose the molting larvae to white light [139] to ensure larvae migration from the egg shells to the sides of the bottle.
(C) Randomly select one bottle of larvae from the corresponding rabbit within treatment groups to be used in subsequent infestation. Place the other bottles of larvae at −20 °C to freeze sterilize the larvae for at least 3 h. Remove the microtube with egg shells, pour the larvae on a Petri dish, weigh and count the larvae. Make a larvae count for at least three egg batches and calculate the average number of larvae. Then, weigh the other larvae. Based on the proportionality between the average larvae count and the weight of larvae from the remaining eggs, calculate the larvae count per egg batch. Calculate the standard deviation and the average larvae count per group. Given that an equal weight of eggs were incubated to obtain larvae, the average larvae count is representative of all egg clusters. Therefore, it is possible to extrapolate the number of larvae which is used for subsequent infestation.

Step 5: Vaccine effect on larval feeding

Infest the earlier-vaccinated cognate rabbits with larvae hatching from at least one egg batch and determine the engorged larvae recovery. Observe the time taken for the larvae to feed to engorgement. Incubate the recovered engorged larvae under appropriate conditions [137].

Step 6: Vaccine effect on larval molting

After larval molting, count the number of nymphs that were recovered. It is possible to also take note of the time taken for the larvae to molt into nymphs. Incubate the nymphs under the appropriate conditions.

Step 7: Vaccine effect on nymph feeding

Feed the nymphs on the earlier-vaccinated cognate rabbits. Assess the nymph feeding success based on the nymph recovery. Incubate the recovered nymph under appropriate conditions. Afterwards, assess whether the vaccine affects nymph molting.

Step 8: Vaccine effect on adult tick engorgement and egg laying

Repeat Step 1 and Step 2, but with slight modifications. In brief, infest the vaccinated cognate rabbits with 60 ticks. Monitor and assess the following parameters: (1) the attachment success after 24 h and feeding time interval to engorgement; (2) the number of female ticks which feed to engorgement; and (3) the egg laying capacity of the ticks.

Step 9: Determine vaccine efficacy

Calculate the vaccine overall efficacy based on the vaccine-induced effect on different tick biological parameters (e.g., tick feeding, egg laying, egg hatching, larvae and nymph molting).

Although other data may be collected, the key parameters below should be used for assessing anti-tick vaccine efficacy. The rationale is that these are the visible indicators/markers of tick population growth under field conditions.

(A) Assess the vaccine effect on the feeding of female ticks

Determine the standard deviation and mean of female ticks harvested in Step 2 and 8. Using the mean value, calculate the percentage effect on tick feeding.

Therefore, % effect on the feeding of female ticks

$$E_{\mathrm{TF}}\;=\;100\;\times \;[\frac{TFcontrol-TFvaccinated}{TFcontrol}]\;=\;100\;\times \;[1-\frac{TFvaccinated}{TFcontrol}]$$

where *TF_Contro__l_* = mean of ticks from the control group, *TF_Vaccinated_* = mean ticks from the vaccinated group.

(B) Assess the vaccine effect on egg laying

Determine the standard deviation and mean of eggs from the ticks harvested in Step 3 and Step 8. Using the mean value, calculate percentage effect on egg laying.

Therefore, % effect on egg laying

$$E_{\mathrm{TE}}\;=100\;\times \;[\frac{TEcontrol-TEvaccinated}{TEcontrol}]\;=\;100\;\times \;[1-\frac{TEvaccinated}{TEcontrol}]$$

where *TE_Control_* = mean of eggs from tick of the control group, *TE_Vaccinated_* = mean of eggs from ticks of the vaccinated group.

(C) Assess the vaccine effect on egg hatchability

Determine the standard deviation and mean of larvae hatching from eggs harvested in Step 4. Using the mean value, calculate percentage effect on egg hatching.

Therefore, % effect on egg hatchability

$$E_{\mathrm{TH}}\;=\;100\;\times \;[\frac{THcontrol-THvaccinated}{THcontrol}]\;=\;100\;\times \;[1-\frac{THvaccinated}{THcontrol}]$$

where *TH_Control_* = mean of larvae hatched from the control group, *TH_Vaccinated_* = mean of larvae hatched from the vaccinated group.

(D) Assess vaccine effect on larvae feeding

Determine the standard deviation and mean number of engorged larvae harvested in Step 5. Using the mean value, calculate percentage effect on larvae feeding.

Therefore, % effect on larvae feeding

$$E_{\mathrm{TL}}\;=\;100\;\times \;[\frac{TLcontrol-TLvaccinated}{TLcontrol}]\;=\;100\;\times \;[1-\frac{TLvaccinated}{TLcontrol}]$$

where *TL_Control_* = mean of engorged larvae from the control group, *TL_Vaccinated_* = mean of engorged larvae from the vaccinated group.

(E) Assess vaccine effect on nymph feeding

Determine the standard deviation and mean of engorged nymph harvested in Step 7. Using the mean value, calculate percentage effect on nymph feeding.

Therefore, % efficacy on nymph feeding

$$E_{\mathrm{TN}}\;=\;100*[\frac{TNcontrol-TNvaccinated}{THcontrol}]\;=\;100\;*\;[1-\frac{TNvaccinated}{TNcontrol}]$$

where *TN_Control_* = mean of engorged nymph from the control group, *TN_Vaccinated_* = mean of engorged nymph from the vaccinated group.

(F) Calculate vaccine overall efficacy

The vaccine overall efficacy is a product of the vaccine relative effect on different key biological parameters among ticks from the vaccinated (Vac) and control (Cont) group. The parameters are: TF (Tick feeding), TE (egg laying), TH (egg hatchability), TL (larvae feeding), and TN (nymph feeding).

Therefore, percentage overall efficacy (E%):

$$E\%\;=\;100\;\times \;[1-[[\frac{TFvac}{TFcont}]\;\times \;[\frac{TEvac}{TEcont}]\;\times \;[\frac{THvac}{THcont}]\;\times \;[\frac{TLvac}{TLcont}]\;\times \;[\frac{TNvac}{TNcont}]]]\;=100*[1-[ETF*ETE*ETH*ETL*ETN]]$$

### 5.2. Can the Approach Be Used to Assess Vaccine Efficacy against the One-Host Ticks?

This approach could be also used to assess the efficacy of anti-tick vaccines against one-host ticks. However, the difference between the feeding behaviors of the one-host and three-host ticks influences how the method is applied. Unlike three-host ticks, one-host ticks (e.g., *R.* *microplus*, *R. decoloratus*, *R. annulatus*) undergo their entire lifecycle on the same host [140]. Note that, unlike with the three-host ticks, one-host ticks do not successfully feed on rabbits [135] or even on other small animal models such as guinea pigs, mice, and rats. Rather, one-host ticks thrive best on cattle. Considering that cattle are more costly, researchers may consider using sheep or goats given that they are closely related to cattle [141]. Contrary to three-host ticks, assessing vaccine efficacy against one-host ticks should include the number of adults obtained after the initial infestation, the number of eggs laid per tick and the number of hatched larvae per egg batch.

Step 1: Assess vaccine immunogenicity and prepare study ticks

Administer animals with the prime booster vaccine dose. Use ELISA tests to determine vaccine immunogenicity before and during the tick infestation experiments. Select at least 10 engorged colony ticks (of equal weight and from the same batch) and incubate them independently under appropriate conditions to allow egg laying. Select the number of ticks based on the number of animals to be used in the experiment. Determine the average number of eggs laid per tick. For example, using a paper, pool and carefully mix the eggs from all the ticks. Dispense one average weight per tick into independent microtubes and place the microtube in another bottle and incubate under appropriate conditions. After hatching, expose the emerged larvae to white light to enhance and hasten larvae migration [139] from the microtube to the bottle. Determine the number of larvae per average tick egg weight as follows:

Randomly select at least three bottles and keep the bottles at −20 °C for at least 12 h. The aim is to sterilize the larvae. Thereafter, remove the microtube which contains the egg shells, pour out the larvae, weigh and count. Considering that egg batches were of equal count and number, the established average larvae count applies to all the obtained larvae.

Step 2: Assess the vaccine effect on adult tick engorgement

After prime-boost vaccination, infest experimental animals with a predetermined number of larvae, collect and count the self-detached (full or partially engorged) adult female ticks, and weigh each. Note that although the resulting ticks may be of lower weight, this does not imply that such ticks cannot lay or that the eggs will not hatch.

Step 3: Assess vaccine effect on adult tick egg laying (oviposition).

Randomly select 30 engorged female ticks per animal and incubate each tick independently under appropriate conditions. Calculate the average tick number per animal as earlier illustrated in Step 3. Pool and mix the eggs as described in the tick preparation stage (Step 1).

Step 4: Assess vaccine effect on egg hatchability.

After egg hatching, process the emerged larvae per egg batch and count as described in Step 4 (c).

Step 5: Assess vaccine effect on larvae to adult development.

Infest the vaccinated animals with a predetermined number of the hatched larvae. After, assess the tick engorgement, oviposition, and egg hatchability as in Steps 2, 3, and 4.

Step 6: Vaccine efficacy assessment

Calculate the mean of ticks dropping off the host, laid eggs, hatched larvae, and the percentage effect (as described in Step 9, A, B, and C, respectively) on tick feeding (TF), egg laying (TL), and egg hatching (TH).

Therefore, calculate the percentage overall efficacy (E%) as follows:

$$E\%\;=\;100\;\times \;[1-[[\frac{TFvac}{TFcont}]\;\times \;[\frac{TEvac}{TEcont}]\;\times \;[\frac{THvac}{THcont}]]]\;\;=100\;\times \;[1-[ETF\;\times \;ETE\;\times \;ETH]]$$

In comparison to the previous methods [50], the proposed method reduces the number of tasks such as sorting larvae from eggs shells, over infesting an animal, and the subsequent challenge of tick incubation during oviposition. Note, however, that the findings only depict the vaccine efficacy, not effectiveness (the reasons are discussed subsequently)

### 5.3. Foreseen Limitations and Opportunities of the Vaccine Efficacy Assessment Approach

In summary, the above-proposed method examines the potential of anti-tick vaccines to reduce tick populations. However, two questions may arise: (1) during the experiments, will the experimental animals, e.g., rabbits or sheep, not acquire humoral resistance against ticks?, and (2) Will that not affect the vaccine efficacy assessment?

In response, first with reference to vaccine efficacy against three-host ticks, it should be remembered that the life cycle entails ticks feeding at three times and on different vertebrate hosts. This, however, does not indicate that three-host ticks cannot feed on the same host at all stages of the tick life cycle. In other words, the ticks do not have a choice; rather, what matters is acquiring a blood meal for continuity of the life cycle. For instance, under the controlled farm system, there is a high probability that at different stages of the life cycle, the ticks may feed on the same animal. This described scenario is similar to what transpires with one-host ticks that feed on one animal throughout its life cycle.

Against that background, the possibility that the experimental animals will acquire tick resistance cannot be ruled out completely. However, evidence indicates that for an animal to acquire tick resistance, multiple tick infestations are required [142] and that the degree of resistance is directly proportional to the number of infesting ticks. Particularly for the three-host ticks, the degree of resistance also varies depending on the preceding tick stage of feeding [24] and the interval between infestations [142]. Specifically, although the animals may acquire some resistance, this is not likely to significantly affect the vaccine efficacy assessment. The reasons are that in the proposed protocol: (1) for the three-host vaccine efficacy studies, rabbits are infested once with the nymph, larval, and adult stages; (2) for vaccine efficacy studies against one- or three-host ticks, a few ticks are used during feeding; and (3) there is an interval between each stage of feeding as the ticks molt to the next state. Note that to improve of the quality of vaccine efficacy data, prior to the experiment, the animals must be naive to ticks.

Another question may arise, particularly with vaccine efficacy studies against one-host ticks: Will the vaccine efficacy assessment against tick feeding not be skewed given that the number of male and female ticks on the animal is not known? The rationale would be that at the start of the experiment, the researcher cannot determine how many females will molt from the nymphs. Nor can he determine the number of females while they are on the animal. Unfortunately, the information regarding the ratio of female and male tick molting from nymphs from a batch of eggs of one-host ticks is still scarce. For that reason, unless there is information on the male/female tick ratio, the researcher may consider focusing on assessing the vaccine effect on egg laying (TE) and egg hatching (TH). In this case, the protocol can be modified accordingly. By contrast, with the three-host ticks, the researcher determines the exact number of ticks to attach and it is possible to keep track of all the subsequent stages especially if engorged ticks are collected from the animals. In other words, the modification proposed in the vaccine efficacy assessments against the one-host ticks does not apply to assessments against three-host ticks. Fortunately, under lab conditions, it is practically possible to conduct independent investigations to establish the male/female tick ratio per egg batch of one-host or three-host ticks.

Nonetheless, through this approach it is possible to (1) determine the degree of resistance based both on the humoral response of the control rabbits and the effect on ticks, (2) establish the span of the humoral immune response or antibody production following the humoral immune response kinetics curve (Figure 3)—this helps the researcher to further investigate the question on the number of vaccine booster doses required, (3) establish whether the vaccine can induce protection against the different stages of tick development, and (4) establish whether natural immunity augments anti-tick vaccine efficacy.

## 6. Approach to Assessing Vaccine Effectiveness

Often, vaccine ‘efficacy´ is mistaken for vaccine ‘effectiveness´ and vice versa [46], yet the terms are distinct [47]. Vaccine effectiveness is defined as the measure of the vaccine performance under uncontrolled field conditions. An alternative defination is that vaccine effectiveness is the measure of how a vaccine that was earlier tested under ideal experimental house/laboratory condition performs under natural conditions. What this means is, unlike the vaccine efficacy data, the vaccine effectiveness assessment data could influence decision making or policy before the vaccine is launched for public livestock health.

Although steady progress has been made in anti-tick vaccine research [142,143,144], only Bm86 is reported to be effective under field conditions [145,146,147]. Specifically, the reports on the effectiveness of Bm86 anti-tick vaccines were based on basic assessments of tick burden and the prevalence of tick-borne disease (anaplasmosis and babesiosis) infection status among cattle that were consistently vaccinated for at least a decade. Note, however, that under field assessments, the effects of tick predictors such as the Oxpeckers, cattle egrets (*Bubulcus ibis*, *Ardeola ibis*) [2], acaricides [148], or pasture conditions [149] come into play. Such confounding factors are likely to significantly influence the interpretation of data collected for vaccine effectiveness under field clinical trial conditions. Therefore, while assessing the effectiveness of anti-tick vaccines, it is relevant to limit the confounding factors. For instance, a semi-field system has been designed [150] and used for investigating novel malaria mosquito control methods [151]. By contrast, a similar SFS has not been developed for anti-tick vaccine effectiveness assessment clinical trials.

### 6.1. Semi Field System Model for Conducting Anti-Tick Vaccine Effectiveness Studies

**Figure 4:**
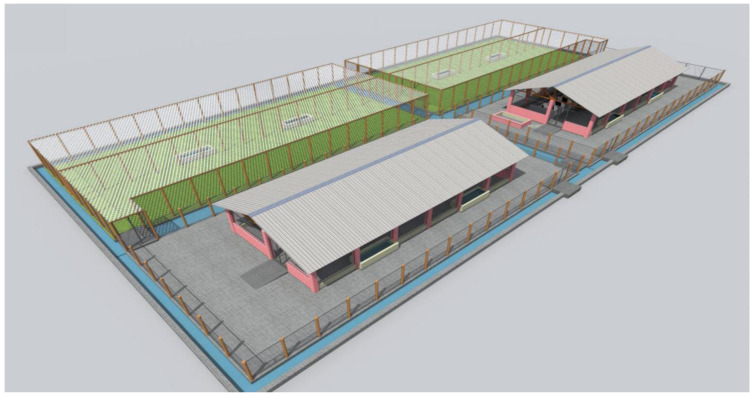
Illustration of the SFS model for conducting anti-tick vaccine effectiveness experiments [154].

As illustrated in Figure 4, the system comprises two cattle shelter houses which can independently accommodate at least 12 cattle. The houses are each surrounded by a trench with water in oil to prevent tick movement from one cattle population to another [52] or from the environment. The water level in the trench is controlled by a safety float value. Adjacent to each house is a paddock with forage that does not inflict anti-tick properties [152,153]. The pasture serves as a breeding ground for ticks, and less so for feeding. Each paddock could be approximately 0.5 hectares. The aim is to limit the cost of fencing for the area (around and above) and increases tick–host encounters. After each tick feeding cycle, the animals should be moved to the houses to allow the grass to grow and ticks to hatch or molt. Experimental animals are returned to the paddocks after sufficient time has passed (depending on the tick life cycle) to allow feeding of the molted ticks.

### 6.2. Assessment of Vaccine Effectiveness

**Figure 5:**
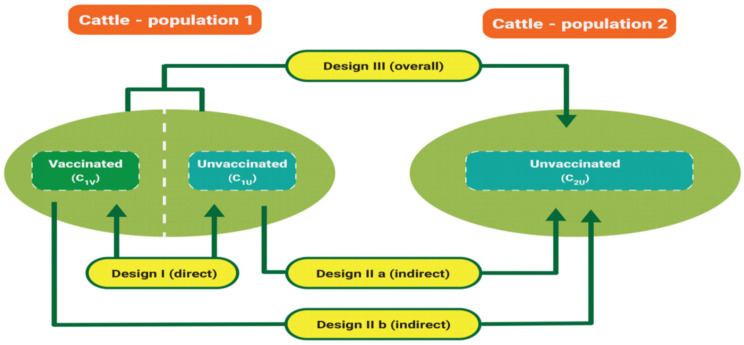
Illustration of the experimental design for assessing anti-tick vaccine effectiveness (adopted from [155]). The assessment is performed using two cattle populations. In Population 1, both the vaccinated (C_1V_) and unvaccinated (C_1U_) cattle are kept together, while in Population 2, only unvaccinated (C_2U_) cattle are present. The two populations are separated from each other. The vaccine-induced effect on ticks from the cattle populations is determined as: Design I: a comparison between effects on ticks from vaccinated and unvaccinated cattle in Population 1. Design II a: a comparison between effects on ticks from unvaccinated cattle in Population 1 and unvaccinated cattle in Population 2. Design II b: a comparison between effects on ticks from vaccinated cattle in Population 1 and unvaccinated cattle in Population 2. Design III: a comparison between effects on ticks from both vaccinated and unvaccinated cattle in Population 1 and unvaccinated cattle in Population 2.

Following the protocol that was used for determining vaccine efficacy above, but with slight modifications below:

(A) Conduct experiments against all ixodid ticks (one-, two-, or three- host tick) using cattle.
(B) In comparison to efficacy studies, conduct vaccine effectiveness studies with a greater number of ticks.
(C) During each stage of tick infestation, collect and handle samples accordingly. This should also aid in monitoring the development of detached field ticks.
(D) After each infestation stage, let the ticks to drop into the pasture and return the cattle to the shelter house.
(E) Assess the vaccine effectiveness using two cattle populations. Figure 5 illustrates the vaccine effectiveness experimental design.

#### 6.2.1. Determine Vaccine Effectiveness

Both anti-tick vaccine efficacy and effectiveness are calculated using the aforementioned formulae [48]. Vaccine effectiveness against three-host ticks assesses the effect on egg laying (TE), egg hatchability (TH), larvae to nymph molting (TL), and nymph to adult molting (TN). By contrast, the assessment of vaccine effectiveness against one-host ticks is based on two parameters: egg laying (TE) and egg hatchability (TH). This is attributed to the differences in tick life cycles. Contrary to the proposed protocol for assessing vaccine efficacy, it is not possible to assess the vaccine effectiveness against tick feeding (TH). The reason is that unlike in the vaccine efficacy assessment, under the vaccine effectiveness assessment, ticks are allowed to drop to the ground into the grass where they will continue their life cycle. In other words, it is not possible to count how many ticks have engorged and dropped onto the grass. Nonetheless, the parameters selected (TE, TH, TL, and TN) are clear indicators of tick burden under field conditions. The assessment is based on the representative tick samples collected from specific cattle in a particular population. After assessing the effect on each parameter, the ticks may be returned to the respective pasture, although not returning the collected samples will not significantly affect the outcome.

Finally, to determine vaccine effectiveness, examine the vaccine-induced effects for at least one year. The objective of conducting vaccine effectiveness studies for at least a year is to incorporate the tick infestation cycles during rainy and sunny season.

The calculations are based on the effect induced on ticks collected from cattle denoted as follows.

C_1U_: Ticks from unvaccinated cattle in population 1.
C_2U_: Ticks from unvaccinated cattle in population 2.
C_1V_: Ticks from vaccinated cattle in population 1.
C_OVR_: Overall tick from vaccinated and unvaccinated cattle population in 1.

#### 6.2.2. Determining Vaccine Effectiveness against Three-Host Ticks

What is the vaccine effectiveness on egg laying?

Collect a predetermined number of engorged adult female ticks from each cattle in a specific population. Based on the mean egg count, determine whether the vaccine affects tick egg-laying ability (for guidance on how to count tick eggs, see the aforementioned mathematical illustrations 5.1: Step 3). The assumption is that after imbibing the vaccine-induced antibodies, ticks are not able to feed to full engorgement, and hence will lay less eggs than ticks which fed on unvaccinated cattle.

Therefore, % effectiveness on egg laying (TE)

Design I (direct)

$$100\;\times \;[\frac{TEC1U-TEC1V}{TEC1U}]\;=\;100\;\times \;[1-\frac{TEC1V}{TEC1U}]$$

Design II a (indirect)

$$100\;\times \;[\frac{TEC2U-TEC1U}{TEC2U}]\;=\;100\;\times \;[1-\frac{TEC1U}{TEC2U}]$$

Design II b (indirect)

$$100\;\times \;[\frac{TEC2U-TEC1V}{TEC2U}]\;=\;100\;\times \;[1-\frac{TEC1V}{TEC2U}]$$

Design III (Overall)

$$100\;\times \;[\frac{TEC2U-TECOVR}{TEC2U}]\;=\;100\;\times \;[1-\frac{TECOVR}{TEC2U}]$$

What is the vaccine effectiveness on egg hatching?

The aim is to assess the whether the vaccine affects egg larvae hatching. The larvae used herein are obtained after incubation of the eggs predetermined above. The assumption is that not all eggs laid by the ticks fed on vaccinated animals will hatch to larvae.

Therefore, % effectiveness on egg hatching (TH)=

Design I (direct)

$$100\;\times \;[\frac{THC1U-THC1V}{THC1U}]\;=\;100\;\times \;[1-\frac{THC1V}{THC1U}]$$

Design II a (indirect)

$$100\;\times \;[\frac{THC2U-THC1U}{THC2U}]\;=\;100\;\times \;[1-\frac{THC1U}{THC2U}]$$

Design II b (indirect)

$$100\;\times \;[\frac{THC2U-THC1V}{THC2U}]\;=\;100\;\times \;[1-\frac{THC1V}{THC2U}]$$

Design III (overall)

$$100\;\times \;[\frac{THC2U-THCOVR}{THC2U}]\;=\;100\;\times \;[1-\frac{THCOVR}{THC2U}]$$

What is the vaccine effectiveness on larvae feeding?

The aim is to assess whether, under field conditions, the vaccine affects larval development. To attain this goal, collect predetermined larvae from the cattle populations, incubate and compare the larval molting success. The assumption is that if the vaccine-induced antibodies affecting larval blood ingestion (e.g., by impeding larval full engorgement), this could reciprocate to reduced success in larval molting.

Therefore, % effectiveness on larval feeding (TL)

Design I (direct)

$$100\;\times \;[\frac{TLC1U-TLC1V}{TLC1U}]\;=\;100\;\times \;[1-\frac{TLC1V}{TLC1U}]$$

Design II a (indirect)

$$100\;\times \;[\frac{TLC2U-TLC1U}{TLC2U}]\;=\;100\;\times \;[1-\frac{TLC1U}{TLC2U}]$$

Design II b (indirect)

$$100\;\times \;[\frac{TLC2U-TLC1V}{TLC2U}]\;=\;100\;\times \;[1-\frac{TLC1V}{TLC2U}]$$

Design III (overall)

$$100\;\times \;[\frac{TLC2U-TLCOVR}{TLC2U}]\;=\;100\;\times \;[1-\frac{TLCOVR}{TLC2U}]$$

What is the vaccine effectiveness on nymph feeding?

The aim is to assess whether, under field conditions, the vaccine affects nymph development. Similarly, collect predetermined nymph from the cattle populations, incubate and compare nymph molting success. The assumption is that if the vaccine-induced antibodies affect nymph blood ingestion (e.g., by impeding larval full engorgement), this could lead to reduced nymph molting success.

Therefore, % effectiveness on nymph feeding (TN)

Design I (direct)

$$100\;\times \;[\frac{TNC1U-TNC1V}{TNC1U}]\;=\;100\;\times \;[1-\frac{TNC1V}{TNC1U}]$$

Design II a (indirect)

$$100\;\times \;[\frac{TNC2U-TNC1U}{TNC2U}]\;=\;100\;\times \;[1-\frac{TNC1U}{TNC2U}]$$

Design II b (indirect)

$$100\;\times \;[\frac{TNC2U-TNC1V}{TNC2U}]\;=\;100\;\times \;[1-\frac{TNC1V}{TNC2U}]$$

Design III (overall)

$$100\;\times \;[\frac{TNC2U-TNCOVR}{TNC2U}]$$

#### 6.2.3. Calculate the Vaccine Overall Effectiveness

This entails calculating the overall vaccine effectivity per design, based on which the vaccine effectiveness on the entire cattle population is determined.

Therefore, the vaccine effectiveness (E)%

Design I effectiveness (E_I_)

$$E_{I}\;=\;100\;\times \;[1-[[\frac{TEC1V}{TEC1U}]\;\times \;[\frac{THC1V}{THC1U}]\;\times \;[\frac{TLC1V}{TLC1U}]\;\times \;[\frac{TNC1V}{TNC1U}]]]$$

Design II a effectiveness (E_II-a_)

$$100\;\times \;[1-[[\frac{TEC1U}{TEC2U}]\;\times \;[\frac{THC1U}{THC2U}]\;\times \;[\frac{TLC1U}{TLC2U}]\;\times \;[\frac{TNC1U}{TNC2U}]]]$$

Design II b effectiveness (E_II-b_)

$$100\;\times \;[1-[[\frac{TEC1V}{TEC2U}]\;\times \;[\frac{THC1V}{THC2U}]\;\times \;[\frac{TLC1V}{TLC2U}]\;\times \;[\frac{TNC1V}{TNC2U}]]]$$

Design III effectiveness (E_III_)

$$100\;\times \;[1-[[\frac{TECOVR}{TEC2U}]\;\times \;[\frac{THCOVR}{THC2U}]\;\times \;[\frac{TLCOVR}{TLC2U}]\;\times \;[\frac{TNCOVR}{TNC2U}]]]$$

Therefore, vaccine overall effectiveness in the semi field system

$$E_{\mathrm{SFS}}\;=\;100\;\times \;[1-[EI\;\times \;EII-a\;\times \;EII-b\;\times \;EIII]]$$

Determining vaccine effectiveness against one-host ticks

The approach described above could also be used for the one-host ticks, but with a slight modification. In this case, calculation is based on egg laying and egg hatching. The rationale is that unlike three-host ticks, during feeding, the nymph and larvae do not drop off the cattle.

Therefore, the vaccine overall effectiveness in the semi field system (E_SFS%_) is derived from the following vaccine effectiveness (E) calculations:

Design I effectiveness (E_I_)

$$100\;\times \;[1-[[\frac{TEC1V}{TEC1U}]\;\times \;[\frac{THC1V}{THC1U}]]]$$

Design II a effectiveness (E_II-a_)

$$100\;\times \;[1-[[\frac{TEC1U}{TEC2U}]\;\times \;[\frac{THC1U}{THC2U}]]]$$

Design II b effectiveness (E_II-b_)

$$100\;\times \;[1-[[\frac{TEC1V}{TEC2U}]\;\times \;[\frac{THC1V}{THC2U}]]]$$

Design III effectiveness (E_III_)

$$100\;\times \;[1-[[\frac{TECOVR}{TEC2U}]\;\times \;[\frac{THCOVR}{THC2U}]]]$$

The vaccine overall effectiveness in the semi-field system

(1) $$\left(E_{\mathrm{SFS}})\;=\;100\;\times \;[1-[EI\;\times \;EII-a\;\times \;EII-b\;\times \;EIII]\right]$$

### 6.3. Foreseen Limitations and Opportunities of the Vaccine Effectiveness Assessment Approach

The limitation to the proposed model is that the experiment is costly in terms acquiring experimental cattle. Yet, in temperate areas, for instance in Africa, it may be difficult to find cattle with no or minimal tick exposure. The merit of conducting vaccine-effectiveness studies under the proposed SFS model is that the researcher is able to examine the potential of the vaccine to confer “herd immunity”. For a deeper insight on “herd immunity”, see the report by John and Samuel [156]. Specifically, the whole principle of anti-tick vaccine-induced herd immunity is that if you inoculate a high percentage of cattle with an anti-tick vaccine, you will reduce the subsequent tick infestation burden, but also prevent ticks from infesting unvaccinated cattle. Ultimately, a reduction in infestation tick burden reciprocates into low transmission of tick-borne pathogens, and hence a reduction in tick-borne diseases. Furthermore, using the SFS model, it is possible to concurrently assess the vaccine effectiveness against multiple tick species which is a common phenomenon in Africa, e.g., in Uganda.

## 7. A Pipeline/Map for Development of Anti-Tick Vaccines

**Figure 6:**
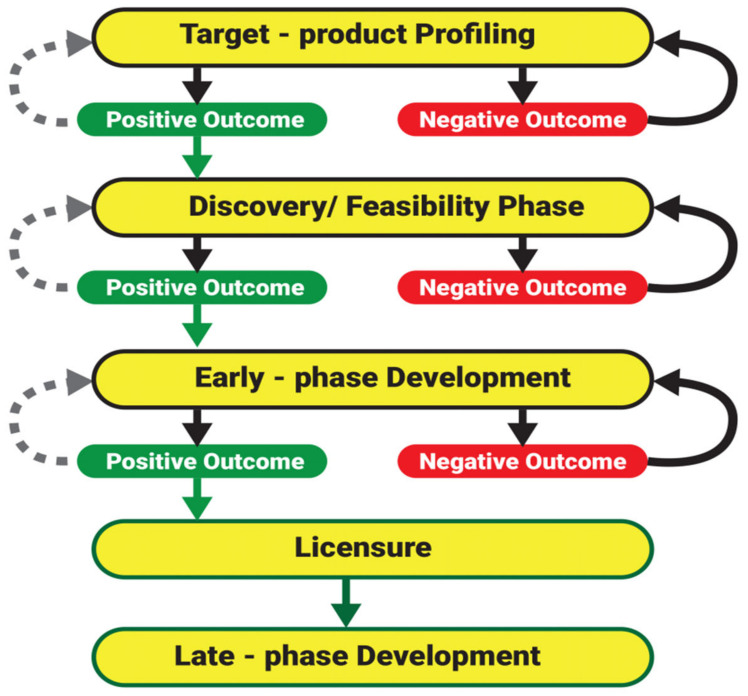
A flow chat for anti-tick vaccine development. Depending on whether the outcome is positive or negative, the phase studies may be repeated as shown in the curved arrow.

Even though a map/pipeline to guide in the development of general veterinary vaccines is established [157], until now, there has been no defined pipeline specific for the development of anti-tick vaccines globally [54]. So far, the available pipeline was based on steps for anti-tick vaccine development in Thailand [46]. By contrast, however, the pipeline described by Jittapalapong et al. [46] is far less elaborate than that presented by Francis [157], and it specifically guides development of a vaccine against *R. microplus*. Therefore, building on the existing pipelines [46,157], and taking into account the bottlenecks in assessing vaccine efficacy and effectiveness, a pipeline (Figure 6) is proposed to guide the development of anti-tick vaccines against one-host and multiple-host ticks.

### 7.1. Phase I: Target Product Profiling

Similarly, this is the first phase for the development of veterinary vaccines. In this case, though, the phase mainly entails: (1) development of the desired product following the basic steps: gene identification/antigen discovery [55]—the steps include: cloning, expression, and protein purification; (2) assess the biochemical functionality of the protein to ensure proper structural conformation which can influence immunogenicity [88]; and (3) assess the immunogenicity of the candidate vaccine. The main aspects of concern include the adjuvant vaccine formulation, dose concentration, vaccination interval, route of administration, optimum or minimum target, and the number of booster doses. Immunogenicity studies can be conducted using small animal models such as rabbits, guinea pigs, and mice. The generated data will guide the subsequent phase studies and it is crucial for product registration.

### 7.2. Phase II: Discovery/Feasibility Studies

This precedes establishment of the vaccine formulation. Specifically, the aim is to show (1) proof of concept of the antigen immunogenicity, and (2) anti-tick vaccine efficacy. Although rabbits are often used due to their immunocompetence [127], they are not compatible hosts for all tick species. For example, the recovery of *R. microplus* ticks fed on rabbits is low [136]. Therefore, to ensure consistency, phase II studies against either one or three-host ticks should be conducted using a universal model, preferably a large animal model. In principle, cattle are the best model; however, they are costly; hence, sheep or goats could be adopted as an alternative. The reason is that in comparison to cattle, sheep and goats are less expensive, yet they are closely related to cattle [81,141]. For example, Jackson and Opedeeck [81] demonstrated the option of using sheep as an alternative model for examining vaccine efficacy. It is also important that the efficacy correlates with the induced humoral response, and in this way an optimum vaccination strategy can be developed. Although the immune response in sheep or goats could vary slightly from that of cattle, the data can demonstrate that the vaccine is suited for vaccine effectiveness assessment. Additionally, the data can suggest that the vaccine could confer protection to sheep or goats especially as both species are hosts under field conditions for some stages of the tick life cycle. Again, note that the vaccine efficacy studies do not substitute vaccine effectiveness studies. Therefore, high vaccine efficacy does not warrant field success.

### 7.3. Phase III: Early Phase Development

This is conducted to establish whether the vaccine can effectively induce protection in cattle. Firstly, following good laboratory practice (GLP), the pre-master seed (vaccine trial batch) is prepared and safely stored. It is important that the source material required for preparation of the antigen is traceable.

Secondly, unlike other vaccines (where the vaccine effectiveness studies are conducted directly in the field), the effectiveness of anti-tick vaccines should be investigated under a semi field system (SFS) (Figure 5). The merits of using this system have been discussed. The other aspects examined under the vaccine effectiveness studies are vaccine biosafety (e.g., antibody traces in milk), variation of immune response (according to animal age, breed, and in pregnant cattle), vaccine adverse effects (e.g., autoimmune response), and immune response duration.

A question may arise whether it is necessary to conduct vaccine effectiveness studies in different geographic areas which would be ideal. However, it would be costly to establish SFS in different geographic regions. Rather, the suggestion is to collect and assess ticks from different regions on one trial site. However, this may also prove costly in terms of the number of cattle required per experiment. Nonetheless, at least one phase-III trial should reflect the vaccine protective effect against ticks from different regions. Before vaccination, it is important to ensure that the cattle are naive to ticks and free from tick-borne diseases and other diseases, as ticks are capable of harboring a plethora of pathogens including bacteria, viruses, fungi, and protozoa [1,2]. If present, the pathogens could be transmitted between cattle during the study. On the other hand, the ticks used in the phase-III studies should be pathogen free. In the event that diseases manifest in experimental cattle, for ethical reasons, these should be immediately treated; however, no acaricides should be applied in the study.

### 7.4. Licensure

On the basis of data generated under Phase I, II and III, an application for vaccine licensure can be compiled and submitted. Furthermore, this data may be sufficient to address the key elements of the regulatory dossier. Upon licensure, upscale vaccine production and commercialization may ensue.

### 7.5. Phase IV: Post Licensure/Late Phase Development

Although these studies precede licensure, they are a continuation of phase III studies. On the contrary, the phase IV studies should be conducted directly under field conditions, such as on farm vaccine trials. Again, the aim is to ascertain the effectiveness of the vaccine in question. In this case, an application to conduct field trials should be submitted to the national drug regulatory body. The application is supported by phase III data. While conducting phase IV studies, it important to further examine aspects such as vaccine biosafety, duration of the humoral immune response, and vaccine safety. For ethical reasons, one may consider conducting the study against one population which consists of vaccinated and unvaccinated cattle. Under these studies, it also important to assess tick-borne disease burden among the study cattle population. However, in the event that tick-borne diseases manifest among experimental cattle, for ethical reasons, these should be immediately treated. However, no acaricides should be applied in the study. An alternative is to use recombinant antigens to not-live pathogens to vaccinate cattle against relevant tick-borne diseases prior to commencement of the trial.

## 8. Concluding Remarks

Currently, although advances has been made in identifying tick antigens, far less effort has been devoted to exploring the immunological related factors that influence vaccine efficacy. Furthermore, the methods for determining vaccine efficacy are still questionable, and there is not a defined protocol for assessing anti-tick vaccine effectiveness.

The approaches proposed herein for assessing vaccine efficacy or effectiveness aims to complement earlier methods [50,51], and to arouse the curiosity of tick researchers concerning how efficacy and effectiveness evaluation studies could be executed while taking into account the ultimate question: can the anti-tick vaccines reduce the tick population? Additionally, given the differences in tick life cycles, it could be erroneous to directly apply the formula used to determine vaccine efficacy or effectiveness against one-host ticks for vaccine assessment against three-host ticks.

The bottlenecks to efficacious anti-tick vaccines discussed herein and the proposed pipeline do not only apply to ixodid anti-tick vaccines, but also in determining argasid anti-tick vaccine efficacies. However, on the contrary, the model proposed for determining anti-tick vaccine efficacy and effectiveness suits mainly ixodid ticks, but not the argasid ticks. This is attributed to the difference between the ixodid and argasid tick life cycles [4].

Similar to development of other veterinary vaccines [157], anti-tick vaccines could take up to 5 to 6 years, yet by contrast, human vaccines require up to 10–20 years [157], except for COVID-19 vaccines. In other words, given the public importance of livestock, tick researchers ought to rigorously scrutinize the candidate anti-tick vaccines before field-application use of anti-tick vaccines. For this reason, a uniform pipeline/map to assist in assessing the efficacy and effectiveness of anti-tick vaccines globally is urgently needed as earlier highlighted at the CATVAC meeting [54]. Fortunately, for instance, the semi-field system model for vaccine effectiveness studies (Figure 5) proposed herein is simple, practical, and minimizes the number of animals. Yet, it helps the researcher to fast-track the potency of the vaccine compared to when the studies are directly conducted in the field.

With slight modifications, the pipeline could also be followed, in assessing the efficacy and effectiveness of cocktail anti-tick vaccines [55] and tick pathogen transmission-blocking vaccines [15]. Furthermore, the factors that influence vaccine humoral immune response induction may also apply towards enhancing the efficacy and effectiveness of cocktail vaccines [55]. The SFS model could also be explored while assessing other methods of tick control, for example, biological and chemical acaricides.
